# Development of a recombinant antibody to target peptides and proteins to sialoadhesin-expressing macrophages

**DOI:** 10.1186/1472-6750-13-33

**Published:** 2013-04-10

**Authors:** Karen Ooms, Hanne Van Gorp, Tim Van Gaever, Hans J Nauwynck, Peter L Delputte

**Affiliations:** 1 Laboratory of Virology, Department of Virology, Parasitology and Immunology, Faculty of Veterinary Medicine, Ghent University, Salisburylaan 133, Merelbeke, 9820, Belgium; 2 Laboratory of Microbiology, Parasitology and Hygiene (LMPH), Faculty of Pharmaceutical, Biomedical and Veterinary Sciences, Antwerp University, Antwerp, Belgium

**Keywords:** Macrophage, Sialoadhesin, CD169, Siglec-1, Recombinant antibody, Targeting, Cell-directed therapy

## Abstract

**Background:**

Sialoadhesin (Sn)-expressing monocytes/macrophages have been associated with several diseases like inflammatory and autoimmune disorders as well as viral infections, and they also appear to play a role in the initiation of an adaptive immune response. This makes Sn-expressing cells not only attractive targets for cell-directed therapies, but also an appealing target for vaccination. Furthermore, since Sn was shown to be an endocytic receptor, the conjugation of effector molecules to an Sn-specific ligand should allow intracellular delivery of these conjugates. Previously, we developed functional Sn-specific immunoconjugates that were generated via chemical coupling. Although successful, the system requires significant optimization for each immunoconjugate to be made. To generate a more flexible and controlled system, we developed a recombinant antibody vector allowing the creation of genetic antibody fusion constructs. This paper reports on the characterization of the recombinant antibody and the evaluation of its use for Sn-directed targeting.

**Results:**

The variable domains of the porcine Sn-specific monoclonal antibody 41D3 were sequenced and cloned in frame with a mouse IgG1 backbone. Transfection of HEK293T cells with the resulting plasmid led to the secretion of fully assembled IgG into the culture medium. This recombinant antibody rec41D3 was shown to specifically bind to porcine Sn with a comparable affinity as the native monoclonal antibody. In addition, rec41D3 also induced Sn endocytosis in primary macrophages and resided for prolonged times in early/late endosomes. To allow the generation of antibody fusion constructs, a multiple cloning site was introduced at the C-terminus of the heavy chain. Two fusion constructs were generated, one containing a V5 peptide tag and one containing an eGFP molecule. Both constructs were shown to be efficiently produced in HEK293T cells and easily purified using standard protein G chromatography. In addition, both V5 and eGFP were shown to be co-internalized together with rec41D3 into Sn-expressing primary macrophages.

**Conclusions:**

A recombinant antibody allowing targeted delivery of peptides and proteins to Sn-expressing macrophages was developed. Production and purification of antibody fusion constructs was possible without major optimization and with batch to batch consistency, confirming the development of a versatile antibody vector to evaluate Sn-directed targeting strategies in a porcine animal model.

## Background

Sialoadhesin-expressing (Sn^+^) macrophages have gained increased attention lately because of their unique distribution in lymphoid organs and their redistribution upon immune activation [1]. Situated in the spleen and other secondary lymphoid tissues, Sn^+^ macrophages appear to be strategically placed for antigen acquisition and delivery to lymphocytes. Junt et al. showed that in lymph nodes, Sn^+^ macrophages capture viral particles (vesicular stomatitis virus) within minutes after subcutaneous injection, transport them across the subcapsular sinus floor and present them to migrating B cells in the underlying follicles [2]. Moreover, also particulate antigen and immune complexes have been shown to be captured and displayed by Sn^+^ macrophages [3-5]. Besides, Sn^+^ macrophages present lipid antigens in a CD1d dependent manner to *i*NKT cells, leading to *i*NKT cell activation and population expansion [6]. Also, Sn^+^ macrophages have been implicated in the activation of CD8^+^ T cells by either directly presenting antigen to CD8^+^ T cells [7] or by transferring the antigen to CD8^+^ dendritic cells in the spleen [8]. Ultimately, several independent research groups showed an enhanced cellular and/or humoral immune response upon Sn-targeted antigen delivery [9-13]. Together, this suggests that Sn^+^ macrophages may act as specialized antigen presenting cells involved in the antigen transport chain and contribute to the growing interest in Sn^+^ macrophages for vaccination strategies, as recently reviewed by Martinez-Pomares and Gordon [1].

Sn (CD169, Siglec-1) is also present on inflammatory macrophages and activated monocytes [14,15]. In affected tissue samples of rheumatoid arthritis patients for instance, high expression of Sn was found on inflammatory macrophages [14]. In addition, abundant Sn expression on inflammatory monocytes/macrophages was shown to correlate with disease severity in pathological conditions like multiple sclerosis, atherosclerosis and breast cancer [16-18]. These observations promote the idea of an Sn-directed cell therapy aimed at elimination or immunomodulation of these cells. Recently, targeted delivery of an anti-TNF-α oligonucleotide to Sn^+^ macrophages resulted in the relief of lupus-like symptoms in mice [19], further showing the potential of Sn^+^ macrophages as a target for immunomodulation. Sn seems thus a promising target for cell-directed therapy, a strategy that is further encouraged by the restricted expression pattern of Sn limiting unwanted side effects. Furthermore, since Sn was suggested to be an endocytic receptor [10,13], the conjugation of antigens, toxins or drugs to an Sn-specific antibody should allow intracellular delivery of these conjugates.

So far, three systems have been used to selectively target Sn^+^ macrophages, namely antibodies, glycan-coated liposomes and cationic agarose hydrogels [9-13,19,20]. While the latter was unintentionally identified as an Sn-targeting system [19], the glycans on the glycan-coated liposomes were specifically designed to be ligands with a high specificity and affinity for Sn [20]. Previously, our research group has made use of an Sn-specific monoclonal antibody (mAb) to generate functional Sn-specific immunoconjugates via chemical coupling [10]. Although successful, this system implies, just like the two other described Sn-targeting systems, that the cargo to be targeted is independently produced and purified, which translates into significant optimization for each separate conjugate. Also, chemical coupling to antibodies depends on the presence and distribution of reactive groups, e.g. primary amines on lysine residues, that can be located in or near the antigen-binding region. Upon coupling, this might result in partial or complete loss of the antibody’s capacity to bind to the target antigen. In addition, there is limited stoichiometric control because of the large number of reactive groups present in an antibody, leading to a heterogeneous mixture that makes batch to batch consistency hard to effectuate [21]. To overcome these problems associated with chemical conjugation of a cargo to an Sn-specific mAb, we report here on the development of a versatile recombinant antibody vector that allows easy production and purification of defined genetic antibody fusion constructs. The obtained vector will allow us to generate functional antibody-cargo constructs to evaluate Sn-targeting strategies in a porcine animal model.

## Results

### Development of a sialoadhesin-specific recombinant antibody, rec41D3

**Figure 1:**
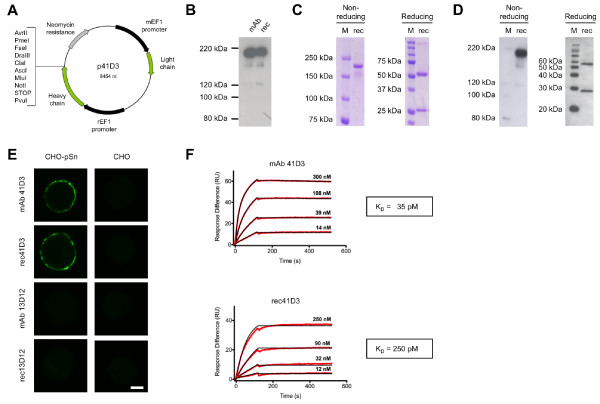
**Development, production and purification of a pSn-specific recombinant antibody, rec41D3.** (**A**) Vector map of p41D3 (**B**) Non-reducing Western blot analysis of hybridoma supernatant of mAb 41D3 compared to p41D3-transfected HEK293T cell supernatant using polyclonal antibodies specific for mouse immunoglobulins (**C**) SDS-PAGE of protein G purified transfected cell supernatants to evaluate rec41D3 purity (**D**) Western blot analysis of purified rec41D3 using polyclonal antibodies specific for mouse immunoglobulins (**E**) Confocal microscopical analysis of CHO cells and recombinant pSn expressing CHO cells (CHO-pSn) incubated with mAb 41D3, rec41D3 or isotype matched controls mAb 13D12 and rec13D12 at 4°C for 1 hour. Cells were fixed and stained with FITC-labelled goat-anti-mouse IgG. Images represent a single confocal z-section through the middle of the cell. Scale bar: 5μm (**F**) Kinetic profile of the pSn-41D3/pSn-rec41D3 interaction by Surface Plasmon Resonance measurements. Immobilized pSn4D-Fc was flowed with rising concentrations of mAb 41D3 or rec41D3, after which injection was halted and dissociation was monitored during 5 minutes. Sensorgrams were fitted to a bivalent model and an approximation of the equilibrium dissociation constant was calculated. M, protein marker; mAb, mAb 41D3; rec, rec41D3; K_D_, equilibrium dissociation constant.

To allow production and purification of genetic fusion proteins of an Sn-specific mAb and other peptides/proteins, a recombinant Sn-specific mAb was made. mAb 41D3 was previously described to selectively bind porcine Sn (pSn) and to be internalized into pSn-expressing macrophages [10]. The variable domains of mAb 41D3 were sequenced and cloned in frame with a mouse IgG1 backbone. The IgG1 backbone was modified so that the resulting plasmid p41D3 contains a C-terminal heavy chain multiple cloning site, allowing removal of the antibody’s heavy chain stop codon and insertion of protein encoding sequences (Figure 1A).

Transfection of HEK293T cells with plasmid p41D3 led to the secretion of a fully assembled IgG into the culture medium of the same size as mAb 41D3, as shown by Western blot analysis of cell culture supernatants using polyclonal antibodies specific for mouse immunoglobulins (Figure 1B). Recombinant antibodies were purified from cell culture medium using protein G chromatography and dialyzed to PBS. To assess the purity of rec41D3 after purification, protein samples were resolved via SDS-PAGE under non-reducing and reducing conditions and Coomassie Blue staining was performed (Figure 1C). The presence of a single band under non-reducing conditions and the presence of two bands under reducing conditions consistent with the sizes of the heavy and light chains of an immunoglobulin showed that protein G purification yields pure rec41D3. Western blot analysis further confirmed that the bands present were antibody light and heavy chain fragments (Figure 1D).

Immunofluorescence stainings of CHO cells expressing recombinant pSn (CHO-pSn, [22]) were performed to assess the specificity of rec41D3 for pSn (Figure 1E). With rec41D3, a bright surface staining, similar to that of the native mAb 41D3, was seen only on CHO-pSn cells, but not on wild type CHO cells, indicating specific pSn recognition of rec41D3. Control stainings were performed with isotype matched irrelevant mAb 13D12 (gD of pseudorabies virus, [23]), and its recombinant form rec13D12. Development, production and purification of rec13D12 was identical to the procedures used for rec41D3.

Since the final goal is to use rec41D3 as a targeting molecule for pSn, the affinity of rec41D3 for pSn is highly important. Therefore, its affinity was determined using Biacore. As a target, a soluble form of the pSn receptor, consisting of the first 4 N-terminal immunoglobulin-like domains of pSn fused to a human IgG Fc (pSn4D-Fc), was coated on the affinity chips. Previously, mAb 41D3 was shown to bind to this pSn4D-Fc [24]. As seen in Figure 1F, both mAb 41D3 and rec41D3 bound with high affinity to pSn4D-Fc, their equilibrium dissociation constants (K_D_) were determined to be 35 and 250 pM respectively.

### rec41D3 induces pSn endocytosis in primary macrophages

**Figure 2:**
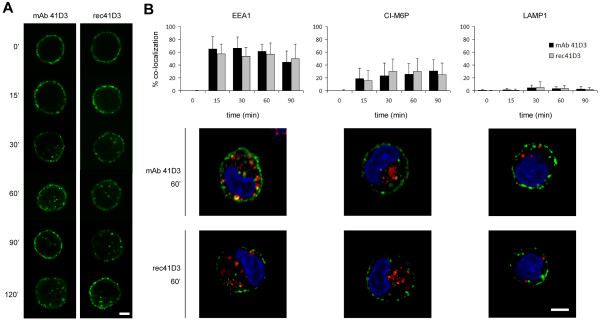
**Analysis of rec41D3-induced pSn endocytosis and analysis of colocalization between internalized antibody and endo/lysosomal compartments.** (**A**) Confocal microscopical analysis of mAb 41D3- and rec41D3-induced pSn internalization in primary macrophages. Cells were incubated for the indicated time periods with either antibody at 37°C, fixed, permeabilized and stained with FITC-labelled goat-anti-mouse IgG. Images represent a single confocal z-section through the middle of the cell. (**B**) Analysis of colocalization between internalized mAb 41D3 or rec41D3 (green) and early endosomes (EEA1), late endosomes (CI-M6P) or lysosomes (Lamp1) (red) in primary macrophages. Colocalization was calculated from confocal z-sections of 25 randomly selected cells of 2 independent experiments at the indicated time points. Data represent the means ± standard deviations. Representative images of macrophages that were incubated for 60 minutes at 37°C with either antibody are shown as overlays of the green and red signal with a yellow colour indicating colocalization. Scale bar: 5 μm.

As our ultimate goal is to target pSn^+^ macrophages *in vivo*, we studied the capacity of rec41D3 to bind pSn and induce its internalization in *in vitro* cultivated primary cells. Primary porcine alveolar macrophages (PAM) were isolated and incubated with the recombinant antibody for different time periods, after which they were fixed and stained to visualize membrane-bound and internalized antibodies. As for mAb 41D3, a clear membrane staining was observed at time zero, while with increasing time, pSn-positive endocytic vesicles became readily apparent (Figure 2A). Also, at early time points, endocytic vesicles of both antibodies were mainly present in the vicinity of the plasma membrane, while with increasing time endocytosed pSn was also localized closer to the perinuclear region. Similar to mAb 41D3-induced pSn endocytosis, rec41D3-induced pSn endocytosis is only partial, as confocal microscopical analysis showed that a clear membrane staining remains visible besides the endocytic vesicles. As a control, PAM were incubated with irrelevant, isotype matched mAb 13D12 and rec13D12. No cell staining was observed with these antibodies (data not shown).

In a previous study, we have shown that mAb 41D3 resides for prolonged times in early endosomes [10]. To analyze the intracellular localization of internalized rec41D3 in comparison to mAb 41D3, double immunofluorescence stainings were performed with EEA1, CI-M6P or Lamp1, markers for early endosomes, late endosomes and lysosomes respectively. For both antibodies, the majority of internalized antibody was localized to early endosomes (around 60%, Figure 2B), while the remainder was localized to late endosomes. Occasionally, a very limited number of internalized antibodies were localized in a lysosomal compartment. These results show that rec41D3 follows an endocytic pathway similar to mAb 41D3 and resides for prolonged times in early/late endosomes.

### rec41D3 targets its cargo V5 as well as eGFP towards pSn^+^ cells

**Figure 3:**
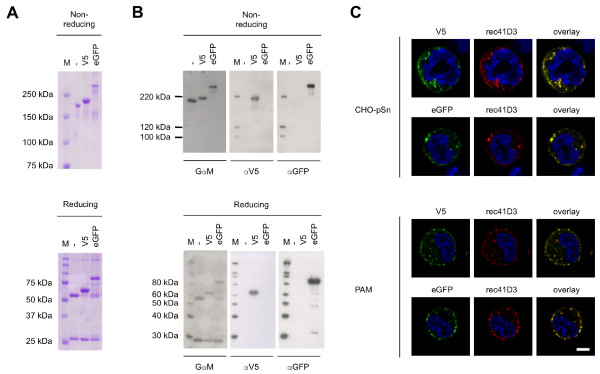
**Development of fusion constructs rec41D3-V5 and rec41D3-eGFP and analysis of their internalization into pSn-expressing cells.** (**A**) SDS-PAGE analysis of protein G purified rec41D3-V5 and rec41D3-eGFP constructs in comparison to unmodified rec41D3 (**B**) Western blot analysis of rec41D3-V5 and rec41D3-eGFP samples in comparison to unmodified rec41D3 using mouse immunoglobulin-specific goat polyclonal antibodies (GαM), a V5-specific mAb (αV5) and an eGFP-specific mAb (αGFP). (**C**) Confocal microscopical analysis of rec41D3-V5 and rec41D3-eGFP internalization in CHO-pSn cells and primary macrophages (PAM). Cells were incubated with rec41D3-V5 or rec41D3-eGFP for 1 hour at 37°C, fixed, permeabilized and stained with AF594-labelled goat-anti-mouse IgG1 in addition to FITC-labelled mouse anti-V5 (IgG2a) or rabbit anti-GFP in combination with FITC-labelled goat-anti-rabbit polyclonal antibodies respectively. Since the rec41D3-eGFP fluorescence was weak, the signal was enhanced to obtain good microscopical images. Images represent a single confocal z-section through the middle of the cell. M, protein marker; -, rec41D3; V5, rec41D3-V5; eGFP, rec41D3-eGFP. Scale bar: 5μm.

The previous results clearly show that rec41D3 can be used to target pSn-expressing macrophages. To be able to evaluate targeting of a cargo, we aimed at generating functional antibody fusion constructs in which a cargo is coupled to the C-terminus of the heavy chain of the antibody. During the generation of the rec41D3 plasmid, a multiple cloning site was introduced at the C-terminus to facilitate this. Also, a flexible glycine-serine (GS) linker [25] was introduced between the C-terminal Fc part and the cargo linked to rec41D3. This minimizes the risk that unwanted interactions occur between rec41D3 and its cargo, which could result in non-functional antibody and/or cargo. Two fusion constructs were generated: one construct containing a (G_4_S)_2_ linker fused to a V5 peptide tag (rec41D3-V5), the other one containing a (G_4_S)_4_ linker fused to eGFP (rec41D3-eGFP). The GS linker used to generate rec41D3-eGFP is long, to ensure eGFP has the opportunity to fold into a functional protein. HEK293T cell transfection followed by protein G purification of the supernatant clearly predominantly yields intact fusion proteins as shown by SDS-PAGE (Figure 3A). Under non-reducing conditions a single band was visible for both fusion proteins, shifted in size in comparison to unmodified rec41D3. Under reducing conditions, it became clear that the antibody’s light chains remained unchanged, while the heavy chains were shifted in size, showing the acquisition of extra protein sequences. The presence of V5 or eGFP at the heavy chains of rec41D3 was further confirmed by Western blot analysis (Figure 3B). These results indicate that rec41D3-V5 contains a V5 peptide tag, while rec41D3-eGFP contains an eGFP molecule.

To further analyze whether or not rec41D3-V5 and rec41D3-eGFP carry their cargo towards pSn^+^ cells and induce internalization into the cells, we incubated CHO-pSn and PAM with the fusion constructs for one hour at 37°C. As shown in Figure 3C, both V5 and eGFP were co-internalized together with rec41D3 in both cell types. As a control, CHO-pSn and PAM were incubated with irrelevant, isotype matched rec13D12 fusion constructs rec13D12-V5 and rec13D12-eGFP. No staining was observed with these constructs (data not shown). In conclusion, these data show that rec41D3 allows the targeting of peptides and/or proteins towards pSn^+^ cells.

## Discussion

Monoclonal antibodies and derivatives are currently the fastest growing class of therapeutic molecules [26]. Their inherent promise to minimize side effects by selectively targeting specific target cells has fuelled their development, leading to several FDA-approved antibody therapeutics so far and many more in the pipeline. Although unmodified mAbs proved their worth, the conjugation of effector molecules (like toxins, drugs, radionuclides,…) to mAbs broadened their therapeutic potential, especially in the domain of cancer therapeutics. Besides cancer, other diseases could also benefit from antibody-directed therapies, the only prerequisite being the identification of a receptor exclusively expressed on those immune cells involved in the induction of pathology. In this respect, sialic acid-binding immunoglobulin-like lectins (siglecs) are compelling candidates for therapy, as they display very restricted expression patterns on subsets of immune cells and may regulate immune cell functions. Furthermore, siglecs are endocytic receptors allowing therapeutic agents conjugated to a mAb to be carried efficiently into the cell [27,28].

Sialoadhesin or Siglec-1 is expressed on cells of the monocyte/macrophage lineage, notably on subsets of resident tissue macrophages and inflammatory monocytes/macrophages [15,27]. A recent report describes the expression of Sn on human mature DCs treated with LPS *in vitro*[29], suggesting that Sn may be present on mature DCs during inflammation *in vivo* as well. Not only have Sn^+^ monocytes/macrophages been described in several diseases like inflammatory and autoimmune disorders as well as viral infections, they also appear to play a role in the initiation of an adaptive immune response as recently shown by different independent research groups and nicely reviewed by Martinez-Pomares and Gordon [1]. Together, this makes these Sn^+^ cells not only attractive targets for cell-directed therapies, but also an appealing target for vaccination. In our previous study, we developed immunoconjugates by the chemical linkage of the model antigen HSA or a toxin to the pSn-specific mAb 41D3 [10]. Although these immunoconjugates proved efficient for boosting immune responses and killing pSn-expressing cells respectively, the chemical linkage of the cargo to a targeting antibody has many disadvantages. First of all, chemical coupling procedures rely on the presence and distribution of reactive groups, like e.g. primary amines on lysine residues, that can be located in or near the antigen-binding region, which upon coupling might result in partial or complete loss of the antibody’s affinity for the target antigen. Secondly, because of the large number of reactive groups present in antibody molecules, a typical distribution can be observed of zero to eight molecules per antibody [30,31], resulting in high variation of the final conjugate. This variation is unwanted, as it may lead to a heterogeneous mixture of components with distinct affinities, stabilities, pharmacokinetics, efficacies, and safety profiles [21]. Moreover, chemical coupling implies that both antibody and cargo to be linked are independently produced and purified, which represents a significant challenge, especially when the cargo is also a biologic. To circumvent these problems, we opted to generate a recombinant form of the pSn-specific mAb 41D3. As shown in this study, this recombinant antibody displays a comparable affinity for pSn compared to the native mAb. In addition, the recombinant mAb also induces pSn endocytosis in primary macrophages, a feature important to allow functionality of antibody-cargo constructs. As protein sequences are attached to the C-terminus of the antibody’s heavy chain, they are less likely to hinder antigen binding by the variable immunoglobulin domains. In addition, each heavy chain will contain only one cargo fused to the C-terminal end. This will result in an antibody with 2 cargos in a defined position and a high intra and inter batch consistency. Furthermore, we could purify the antibody-cargo fusion proteins using standard protein G chromatography, which represents a major advantage compared to chemical coupling in which purification is needed for both cargo and antibody before, as well as after chemical conjugation.

In this study we managed to make genetic fusion constructs of a peptide or a protein linked to our recombinant mAb. Obviously, the recombinant antibody vector does not allow to make genetic fusion constructs with chemical compounds. For vaccination strategies however, this limitation is not expected to pose any problems, as most antigens used in vaccines are protein based. One challenge however would be to ensure correct folding of the antigen upon genetic fusion to the antibody and to maintain this fold during purification procedures. Similarly, immunotoxins can be made using the Sn targeting vector. Although the production of immunotoxins in eukaryotic cells has been limited due to potential toxicity to the producing cells, several independent research groups have reported on the successful production of immunotoxins in mammalian cell lines, including HEK293T [32-34]. In case a specific application would require the chemical linkage to an antibody, e.g. when vaccines are based on glyco-epitopes, a recombinant mAb has some major advantages. It allows addition of specific amino acid modifications to the antibody, which will result in site-specific incorporation of drug molecules through chemical linkage yielding batch to batch consistency of antibody-drug conjugates. Examples of such already implemented modifications are the THIOMAB^TM^ technology of Genentech Inc [35] or the methodology of Axup et al. [21].

As our future plans include the use of the developed recombinant antibody to target antigens towards pSn-expressing macrophages *in vivo*, one might be concerned about the immunogenicity of mouse antibodies in pigs. Poderoso *et al.* previously used mouse mAbs as surrogate antigens in pigs to evaluate the role of Sn in the induction of humoral responses and noticed an enhanced anti-mouse antibody response in comparison with a non-targeting isotype control mAb [11]. The induction of anti-mouse antibodies was however low after primary injection of the mAb, only after a booster vaccination antibody titres rose significantly. Previously, we have observed an enhanced anti-HSA antibody response after a single dose vaccination of HSA coupled to mAb 41D3 without adverse clinical effects [10]. Therefore, in our future experiments, we will use a single dose of rec41D3-antigen to evaluate the protective efficacy of antigen targeting to pSn. If further experiments confirm the applicability of this targeting technology, ‘porcinization’ of the recombinant antibody will be examined to enable prime-booster vaccination schedules.

## Conclusions

A recombinant antibody that targets sialoadhesin was developed. In addition, we constructed a vector that allows the genetic linkage of a protein cargo in a defined position at the C-terminus of both heavy chains of a fully assembled antibody. This vector was shown to be versatile, as both a peptide and a more complex, larger protein could be fused. Furthermore, production and purification of the antibody fusion constructs did not require major optimization. In comparison to other Sn targeting strategies (glycan-coated liposomes and cationic hydrogels), the one-step production of the final carrier-cargo product together with the high specificity and affinity of the recombinant antibody may be considered an advantage for drug development. Future research will mainly focus on the development of functional antibody-antigen fusions allowing the evaluation of Sn-directed vaccination strategies.

## Methods

### Ethics statement

The experimental procedure for the collection of porcine alveolar macrophages was authorized and supervised by the Ethical and Animal Welfare Committee of the Faculty of Veterinary Medicine of Ghent University.

### Cell culture and monoclonal antibodies

PAM were isolated from 4- to 6-week-old conventional Belgian Landrace pigs as described [36], and cultivated in RPMI-1640 medium supplemented with 10% heat-inactivated FBS, 2 mM L-glutamine, 1% non-essential amino acids and 1 mM sodium pyruvate. HEK293T were grown in DMEM medium supplemented with 10% heat-inactivated FBS, 2 mM L-glutamine and 1 mM sodium pyruvate. CHO-K1 cells and CHO-K1 cells stably expressing recombinant pSn [22] were cultivated in F-12 medium supplemented with 10% FBS and 1 mM sodium pyruvate. All culture media were supplemented with a mixture of antibiotics and cell cultures were kept in a humidified 5% CO_2_ atmosphere at 37°C.

MAb 41D3, directed against pSn [37,38], and isotype matched (IgG1) control mAb 13D12, directed against pseudorabies virus glycoprotein gD [23] were purified from hybridoma supernatants using protein G sepharose column chromatography (GE Healthcare), dialyzed to PBS and stored at −70°C until use.

### Construction, production and purification of rec41D3, rec13D12 and fusion constructs

The variable domains of mAb 41D3 were sequenced and cloned into the pVITRO1-neo-mcs vector (Invivogen), in frame with a mouse IgG1 backbone (Fusion Antibodies Ltd). For the construction of rec13D12, total RNA from 10^7^ hybridoma cells was isolated (RNeasy® mini kit, Qiagen) and cDNA was synthesized by RT-PCR (Superscript® III First-Strand Synthesis System, Life technologies). DNA sequences of mAb 13D12 VH and VL were obtained based on the protocol described by the Mouse Ig-Primer Set of Novagen®. Subsequently, the variable domains of rec41D3 in pVITRO1-neo-mcs were exchanged by the variable domains of mAb 13D12, yielding a plasmid encoding rec13D12.

To construct both rec41D3- and rec13D12-V5, a (G_4_S)_2_-V5 DNA sequence was introduced at the 3^′^ end of the heavy chain sequence using forward primer 5^′^-AAACGATCG*GGCGGGGGAGGCTCAGGGGGAGGC;GGGAGC*GGTAAGCCTATCCCTAACCCTCTCCTCGGTCTCGATTCTACGGCGGCCGCATGAACGCGTAAA-3^′^ and reverse primer 5^′^-TTTACGCGTTCATGCGGCCGCCGTAGAATCGAGACCGAGGAGAGGGTTAGGGATAGGCTTACC*GCTCCCGCCTCCCCCTGAGCCTCCCCCGCC*CGATCGTTT-3^′^. To construct both rec41D3- and rec13D12-eGFP, a (G_4_S)_4_ DNA sequence was introduced first at the 3^′^ end of the heavy chain sequence using forward primer 5^′^-AAACGATCG*GGCGGGGGAGGCTCCGGGGGAGGCGGGTCTGGAGGCGGGGGAAGTGGCGGGGGAGGCTCA*GCGGCCGCAAA-3^′^ and reverse primer 5^′^- TTTGCGGCCGC*TGAGCCTCCCCCGCCACTTCCCCCGCCTCCAGACCCGCCTCCCCCGGAGCCTCCCCCGCC*CGATCGTTT-3^′^, followed by the eGFP sequence of pCeMM CTAP(SG) (GenBank Accession number EF467048) amplified by PCR using forward primer 5^′^- AAAGCGGCCGCAATGGTGAGCAAGGGCGAGGAG-3^′^ and reverse primer 5^′^- AAAGGCCGGCCTTACTTGTACAGCTCGTCCAT-3^′^. Underlined sequences represent enzyme restriction sites, sequences in italics represent linker DNA sequences.

For production of all recombinant antibodies and fusion constructs, HEK293T cells were transiently transfected using calcium phosphate. Transfected cells were cultured in DMEM supplemented with 10% IgG depleted, heat-inactivated FBS, 2 mM L-glutamine, 1 mM sodium pyruvate, and a mixture of antibiotics in a humidified 5% CO_2_ atmosphere at 37°C. Culture supernatant was collected and IgG were purified from the supernatant using standard protein G sepharose chromatography following the manufacturer’s instructions (GE Healthcare). Fractions of the eluate containing the purified protein were pooled and the buffer was exchanged to PBS by dialysis. Purified protein was stored at −70°C until use. Yields of recombinant antibodies and fusion constructs from HEK293T supernatant were ± 500 μg/l for rec41D3, rec13D12 and V5 fusion constructs and ± 250 μg/l for eGFP fusion constructs.

### SDS-PAGE, Coomassie blue staining and western blot analysis of rec41D3 and fusion constructs

Samples of purified proteins were mixed with (non-) reducing Laemmli buffer, boiled for 5 min and subjected to SDS-PAGE (6% non-reducing gel, 10% reducing gel) using a BioRad Mini Protean 3 system. For Coomassie Blue staining, the SDS-PAGE gel was incubated successively in Ultra Pure water, Imperial™ protein staining solution (Thermo Scientific) and Ultra Pure water as destaining solution. Alternatively, for Western blot analysis, proteins were transferred from the SDS-PAGE gel to a PVDF membrane (Membrane Hybond-P, GE Healthcare) via Western blotting (BioRad Mini Trans Blot). The membrane was blocked overnight in PBS + 0.1% Tween 20 + 5% skimmed milk. Detection of recombinant antibodies was performed by subsequent incubation of the blot with peroxidase-labeled polyclonal goat anti-mouse antibodies (Dako), followed by visualization using enhanced chemiluminiscence (ECL; GE Healthcare). Alternatively, rec41D3-V5 protein was detected using peroxidase-labeled anti-V5 antibodies (Life technologies) and rec41D3-GFP protein was detected using a recombinant rabbit monoclonal GFP-specific antibody (ABfinity™, Life technologies) and peroxidase-labeled polyclonal goat anti-rabbit antibodies (Dako), followed by ECL visualization.

### Surface plasmon resonance

The generation of a soluble Fc-tagged pSn has been described before [24]. For Biacore experiments, a pSn4D-Fc protein was used containing a single point mutation in the first pSn immunoglobulin-like domain, since production levels of this protein are significantly higher. mAb 41D3 has been shown to equally bind to this protein [24]. After production and purification, pSn4D-Fc was coupled to an activated CM5 chip to 300 responsive units (RU). mAb 41D3 (300, 108, 39, 14 nM) or rec41D3 (250, 90, 32, 12 nM) diluted in HBS-EP buffer was injected (30 μl/min) for 2 min, followed by dissociation for 5 min. Surface regeneration was performed with 10 mM NaOH for 60 s followed by a recovery phase. The sensorgrams were fitted to a bivalent model accounting for mass-transfer effects.

### Immunofluorescence stainings

To assess pSn specificity, CHO or CHO-pSn cells were seeded on poly-L-lysine (Sigma) coated coverslips and incubated with mAb 41D3, rec41D3 or isotype-matched control antibodies mAb 13D12 and rec13D12 for 1 h at 4°C. Cells were then fixed with 4% (w/v) paraformaldehyde (Sigma) in PBS, permeabilized using 0.5% (w/v) saponin (Sigma) in PBS and stained with FITC-labelled goat-anti-mouse IgG to visualize antibodies bound to the cell.

To evaluate pSn endocytosis of rec41D3 in comparison to mAb 41D3, PAM were incubated with 2 μg/200 μl purified antibodies and cells were fixed with 4% paraformaldehyde at the indicated time points. Afterwards, cells were permeabilized using 0.5% saponin and stained with FITC-labelled goat-anti-mouse IgG to visualize antibodies bound to and internalized in the cells. As a control, cells were fixed (time 0) and incubated with the respective antibodies afterwards. For double immunofluorescence staining with markers of the endosomal compartments, the respective primary antibodies for each endosomal compartment followed by appropriate Texas Red-labelled secondary antibodies was used for their visualization. The primary antibodies used were an affinity purified goat pAb (sc-6414; Santa Cruz Biotechnology), a rabbit pAb (ab32815; Abcam) and a rabbit pAb (sc-5570; Santa Cruz Biotechnology), for early endosome antigen 1 (EEA1), cation-independent mannose-6-phosphate receptor (CI-M6P) and lysosome-associated membrane protein 1 (Lamp1) respectively. Cell nuclei were visualized using Hoechst 33342 (Life Technologies).

To evaluate pSn endocytosis of rec41D3 fusion constructs, CHO-pSn and PAM were incubated with 2 μg/200 μl purified rec41D3-V5, rec41D3-GFP or their respective isotype controls rec13D12-V5 and rec13D12-eGFP for 1 h at 37°C. Cells were then fixed with 4% paraformaldehyde, permeabilized using 0.5% saponin and stained with AF594-labelled goat-anti-mouse IgG1 in addition to FITC-labelled mouse anti-V5 (IgG2a) or rabbit anti-GFP in combination with FITC-labelled goat-anti-rabbit polyclonal antibodies respectively. Cell nuclei were visualized using Hoechst 33342 (Life Technologies).

### Confocal laser scanning microscopy

Z-section images of samples were acquired using a Leica TCS SPE-II laser scanning spectral confocal system (Leica Microsystems GmbH) linked to a Leica DM2500 microscope (Leica Microsystems GmbH). Image acquisition was done using the Leica LAS AF confocal software package and analysis of colocalization was done using CoLocalizer Pro [39]. For the colocalization analysis between Sn and the endosomal markers, the Sn-positive plasma membrane was excluded from the analysis and only internalized Sn was considered.

## Abbreviations

Sn: Sialoadhesin; Sn+: Sialoadhesin-expressing; pSn: Porcine sialoadhesin; Siglecs: Sialic acid-binding immunoglobulin-like lectins; mAb: Monoclonal antibody; rec41D3: Recombinant antibody 41D3; rec13D12: Recombinant antibody 13D12; p41D3: Plasmid 41D3; PAM: Porcine alveolar macrophages; GS: Glycine-serine.

## Competing interests

Two authors (Peter L. Delputte and Hans J. Nauwynck) are listed as inventors on a patent application related to the work described in this study, which has been submitted through Ghent University.

## Authors’ contributions

KO participated in the design of the experiments, performed the majority of the experiments and wrote the manuscript. HVG participated in the design of the recombinant control antibody rec13D12 and critically revised the manuscript. TVG carried out the cloning of (G_4_S)_2_-V5 and (G_4_S)_4_-eGFP in rec41D3 and rec13D12 plasmids. PD and HN participated in the design of the experiments, supervised the study and critically revised the manuscript. All authors read and approved the final manuscript.
